# Intensification of Electrochemical Performance of AA7075 Aluminum Alloys Using Rare Earth Functionalized Water-Based Polymer Coatings

**DOI:** 10.3390/polym9050178

**Published:** 2017-05-18

**Authors:** Atzin C. Ferrel-Álvarez, Miguel A. Domínguez-Crespo, Aidé M. Torres-Huerta, Hongbo Cong, Silvia B. Brachetti-Sibaja, Ana B. López-Oyama

**Affiliations:** 1 Instituto Politécnico Nacional, CICATA-Altamira, Grupo CIAMS, Km 14.5 Carretera Tampico-Puerto Industrial Altamira, C.P. 89600 Altamira, Mexico; atzin.ferrel@gmail.com (A.C.F.-Á.); atohuer@hotmail.com (A.M.T.-H.); 2 Department of Chemical and Biomolecular Engineering, Corrosion Engineering Program, Whitby Hall 211, The University of Akron, Akron, OH 44325-3906, USA; hcong@uakron.edu; 3 TecNM, Instituto Tecnológico de Ciudad Madero, D.E.P.I. Ave Primero de Mayo S.N. Col. Los Mangos, C.P. 89449 Cd Madero, Mexico; bbrachetti@hotmail.com; 4 CONACYT Research Fellow–CICATA-Altamira, Ave. Insurgentes Sur 1582, Col. Crédito Constructor, Del. Benito Juárez, C.P. 03940. Cd De Mexico, Mexico; alopezoyama@hotmail.com

**Keywords:** aluminum alloys, AA7075, hybrid coatings, polyurethane, cerium oxide (CeO_2_), nanostructures, coatings, corrosion protection

## Abstract

This work reports the effect of different amounts of ceria nanoparticles on UV resistance and barrier properties of water-based polyurethane (WPU) on glass and AA7075 aluminum alloy substrates. Hybrid coatings were synthesized from an aliphatic WPU–HDI (1,6-hexamethylene di-isocyanate) and cerium oxide nanoparticles (CeO_2_) with an average particle size distribution of about 25 nm. Different nanoceria amounts (1, 3 and 5 wt %), mixing times (30, 60 and 120 min) and methods to disperse the nanostructures into the polymer matrix (magnetic stirring and sonication) were evaluated. Initially, the dispersion of CeO_2_ nanoparticles embedded in the polymer matrix and displacement in the corrosion potential (*E*_corr_) were analyzed by confocal scanning laser microscopy (CLSM) and open circuit potential (*E*_ocp_) measurements. According to this behavior, the dispersion and water ratio required during the polymerization process were established. Coated samples obtained after the second stage were characterized by X-ray diffraction (XRD), scanning electron microscopy (SEM), atomic force microscopy (AFM) and optical light microscopy. In addition, optical measurements on glass substrates were evaluated with UV-vis spectroscopy. The effect of the synthesis parameters on the corrosion behavior of WPU–CeO_2_/AA7075 systems was investigated with *E*_ocp_ and electrochemical impedance spectroscopy (EIS) in a 3 wt % NaCl solution. In addition, the films were subjected to 180 h of accelerated weathering. The results show that the combination of specific nanoceria addition with the optimal synthesis parameters enhances optical transparence of WPU as well as barrier properties. From these, the coated specimens prepared with 3 wt % of ceria content and sonicated for 30 min showed a highly dispersed system, which results in a high charge transfer resistance. The observed properties in clear coats deposited on metallic substrates suggested an improvement in the appearance and less deterioration in UV exposure in comparison with pure WPU, enhancing the protective properties of the AA7075 aluminum alloy when exposed to a corrosive medium.

## 1. Introduction

Polymers are widely used due to the molecular architectures designed for each specific application. From overall polymers, polyurethane (PU) coatings have been successfully used for corrosion resistance, and durability against wear and weathering; in addition, they provide protection of biological attack [1,2]. Waterborne polyurethanes (WPUs) are promising and versatile polymeric materials commonly used in a variety of fields since they are nontoxic, nonflammable and environmentally friendly due to low or no-presence of volatile organic compounds. PUs are synthesized by the reaction of polyols and isocyanates. Different isocyanates such as toluene diisocyanate (TDI), hexamethylene diisocyanate (HDI), isophorone diisocyanate (IPDI), and others are often used for PU synthesis. Among them, HDI is probably one of the most used monomers for polyurethane production and it is commonly employed as top-coat layer for other protective systems on metallic substrates to control corrosion reactions [3,4]. Unfortunately, one of the main disadvantages of PU coatings is the hydroscopic tendency which causes permeation of oxygen or other aggressive ions such as chloride; yellowing caused by the photochemical degradation resulting from ultraviolet (UV) radiation is another disadvantage; both processes greatly cause gradual PU disintegration, affecting its optical and mechanical properties [5,6,7]. Conventional coatings used for corrosion prevention involve zinc/chromate primers, chromate conversion coatings and most recently rare-earth compounds mainly CeO_2_ and La_2_O_3_, whereas hybrid coatings are largely processed with ZnO, SiO_2_ and ZrO_2_ particles [8,9]. Different studies state that the addition of inorganic particles (at nanometric scale) to the PU coatings (hybrid coatings) can improve the mechanical and chemical resistance, favoring the coating appearance [10,11,12,13,14,15,16,17,18,19,20]. Particularly, cerium oxide (ceria, CeO_2_), CeO_2_-coated and CeO_2_-hybrid materials have been extensively investigated in catalysts, sensors, energy storage, UV absorbers and other applications [10,11,12,15,16,17,21]. Cerium oxide nanostructures display high temperature and chemical stability, good electrical contact in comparison with other oxides, and almost the same band gap as TiO_2_ (3.0–3.2 eV), where under UV light, the molecules can be excited to absorb a photon and create an electron–hole pair [22]. In this way, the synergistic effect of organic–inorganic hybrid coatings using rare earth oxides has been proposed as reinforcement to improve the physical barrier against corrosion of different metallic substrates in aggressive media [23,24,25,26,27], where the organic component of the coating provides barrier effect and self-healing properties while the inorganic particles enhance the mechanical strength, chemical resistance and thermal stability [28,29,30]. Herein, we report the synthesis of WPU–CeO_2_ hybrid organic–inorganic coatings in order to enhance the barrier properties of the AA7075 aluminum alloy and avoid yellowing of the system. WPU is proposed as an environmentally friendly alternative to protect aluminum substrates. It is generally known that one of the main challenges faced during the fabrication of organic–inorganic hybrid coatings is to avoid the agglomeration tendency in the polymer matrix due to the superficial high energy of the nanostructures, which can affect negatively the performance in industrial applications. Thus, to reach these goals, a systematic study of ceria content (1–5 wt %), dispersion method (magnetic stirring or sonication) and mixing times (30–120 min) during the synthesis of hybrid coatings was carried out. The effectiveness of the synthesis parameters was analyzed by open circuit potential (*E*_ocp_) and electrochemical impedance spectroscopy (EIS) in chloride medium. Exposure in the salt spray chamber was also evaluated. 

## 2. Materials and Methods

### 2.1. Materials

AA7075 aluminum alloy square plate substrates (20 mm × 20 mm × 1 mm) and glass substrates (75 mm × 25 mm × 1 mm) were used in the present study. The chemical composition of the metallic substrates was analyzed with mass and optical emission spectroscopy using a Spectro Analytical Instrument LAVWA18B, AMETEK, Monterrey, México (Table 1). The following procedure was applied for the metallic substrate preparation: specimens were abraded with successive grades of SiC papers up to 1500-grade (#600, #1000, #1500), washed with a soapy solution, deionized water, ultrasonically cleaned in acetone for 3 min, rinsed with deionized water and blow-dried at room temperature with compressed air. The glass substrates were degreased with a soapy solution, followed by deionized water, sonicated in acetone for 3 min, rinsed with deionized water and blow-dried at room temperature with compressed air.

CeO_2_ nanoparticles in water (10% *v*/*v*) with an average particle size distribution of 25 nm were supplied by Sigma-Aldrich Company (Toluca, México) to assure a proper dispersion study in the polymeric matrix. Commercial water-based polyurethane (WPU HDI) with its corresponding catalyst was supplied by COMEX^®^ (Tepexpan Acolman, México), Series 11000^®^ (Tepexpan Acolman, México), POLYFORM^®^ (Tepexpan Acolman, México).

### 2.2. Synthesis and Deposition of Hybrid Coatings

The synthesis of hybrid materials was carried out using three different weight percentages of HDI (water base) and CeO_2_ to obtain different molar ratios and reach a final PU–CeO_2_ composition of 99:1, 97:3 and 95:5. In the first step, HDI and the proper amount of CeO_2_ nanoparticles were mixed by the sonication method for 30, 60 and 120 min. 

To minimize the temperature increase during the application of the sonication method, it is important to highlight that a procedure using cycles of 10 min on and 1 min off was adopted until total mixing time was reached. Thereafter, a commercial catalyst was added to obtain a 3:1 ratio of WPU–catalyst followed by the spray deposition process onto AA7075 and glass substrates alternating vertical and horizontal orientations. The spray process was repeated up to four times on each substrate in order to obtain uniform wet spray coatings of ~100 μm. The resulting coatings were then dried at room temperature for 24 h, reaching a dried thickness with an average between 30–35 μm, which was measured with a dry film thickness Elcometer inspection equipment (456, for non-ferrous substrates). In the case of the synthesized hybrid coatings using magnetic stirring, a similar procedure was used, although in this case the mixing time was 60 min, as CeO_2_ nanostructures tend to agglomerate with other times.

### 2.3. Characterizaction of Samples

The crystal structure of the dried hybrid coatings and bulk materials were characterized by X-ray diffraction (XRD) with a copper K_α_ αradiation source (λ = 1.5405 Å) at a scan rate of 0.04° min^−1^, using a Bruker AXS D8 Advance apparatus (Bruker, Billerica, MA, USA). The samples were analyzed in the 2θ range from 20° to 80°. Preliminary observations of the hybrid coatings deposited on the AA7075 substrate were performed with a light microscope (OLYMPUS BX51, Olympus Co., Allentown, PA, USA) with magnifications from 5× to 50×.

To evaluate the dispersion degree of CeO_2_ nanoparticles into the polymeric matrix, the hybrid coatings were characterized by confocal laser scanning microscopy (CLSM) using a Carl ZEISS microscope (LSM 700 model, Carl Zeiss AG, Oberkochen, Germany). The measurements were performed considering the emission lines of the system: PU with two emission lines at 627 and 637 nm and 539 nm for CeO_2_ nanoparticles. The topography roughness and profile curves of the as-prepared coatings were characterized by AFM (BRUKER Nanoscope V and BRUKER Dimension Icon Scan Asyst, Bruker, Billerica, MA, USA) at room temperature. The resonance frequency of the tip was 300 kHz, and the scanning rate was 1 Hz. Transmittance spectra of coatings on glass substrates were acquired in the 200–1000 nm range with a scan rate of 600 nm min^−1^, using a Cary UV-Vis-NIR spectrometer (Agilent Technologies, Santa Clara, CA, USA). The average of at least 2 measurements was reported.

The morphological aspect after electrochemical evaluation was examined using the microscope light with magnifications of 10× and scanning electron microscope (SEM) FEI Model Quanta 3D FEG (SEM/FIB), electron back-scatter diffraction (EBSD), FEI Co., Hillsboro, OR, USA, 30 KV and 5 mm.

### 2.4. Electrochemical Characterization

The electrochemical behavior of coated AA7075 was assessed in a 3 wt % NaCl solution at room temperature. The tests were based on the ASTM G3 and ASTM G59 standards [31,32]. A typical three-electrode flat cell was used: the coated AA7075 sheet as the working electrode with an exposed area of 0.785 cm^2^; a saturated calomel electrode (SCE) was the reference electrode and a platinum wire as counter electrode. The following electrochemical techniques were applied using a GAMRY REF600^®^ potentiostat, (Gamry Instrument Inc., Warminster, PA, USA): open circuit potential (*E*_ocp_), and electrochemical impedance spectroscopy (EIS). EIS measurements were carried out at *E*_ocp_ with a sinusoidal potential of 10 mV in amplitude. The frequency range was 10^5^ at 0.01 Hz with 10 points per decade. Each coated sample was exposed to a 3 wt % NaCl solution for 24 cycles. Each cycle consisted of the evaluation of *E*_ocp_ (15 min), EIS (~1 h), and *E*_ocp_ (15 min), which were measured successively up to reaching 24 cycles. Each test was evaluated for about 36 h. In the manuscript, the results are shown at selected cycles, namely the 1st, 12th and 24th cycles. In total, 24 EIS cycles were performed on each specimen. The corrosion behavior of bare (non-coated) AA7075 was also tested under the same conditions, providing a baseline for coating evaluation.

### 2.5. Salt Spray Test

The corrosion resistance of the coatings deposited onto AA7075 was evaluated by salt spray test following instructions of the MIL81706B aeronautical standard [33]. Samples were subjected to a 5% salt spray test in accordance with the ASTM-B117 standard [34] with the exception that the surface of the panels was inclined 6° from the vertical direction. The salt spray tests were conducted in a SINGLETON-SCCH salt spray chamber for 185 h. Bare (non-coated) AA7075 sheets (10 cm × 7.5 cm) were also tested as reference. The samples were comparatively evaluated after the tests.

## 3. Results and Discussion

**Figure 1:**
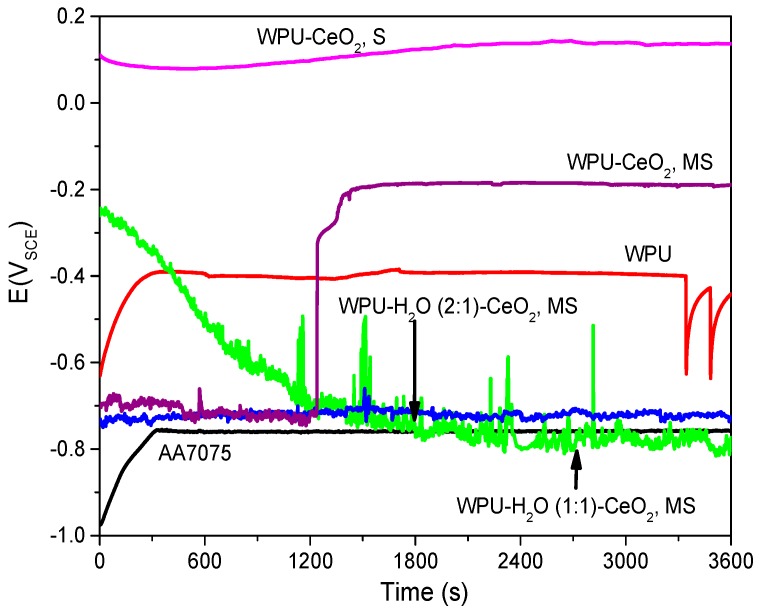
Potential-time behavior of the coated metallic substrates with hybrid coatings using different methods and conditions to disperse 5 wt % of nanoceria into the polymer matrix. Sonication (S) and Magnetic stirring (MS) for 1 h.

**Figure 2:**
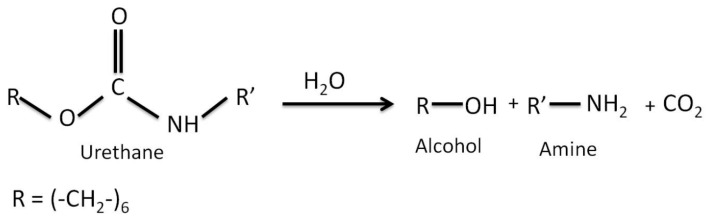
Possible degradation of urethane in the presence of water.

**Figure 3:**
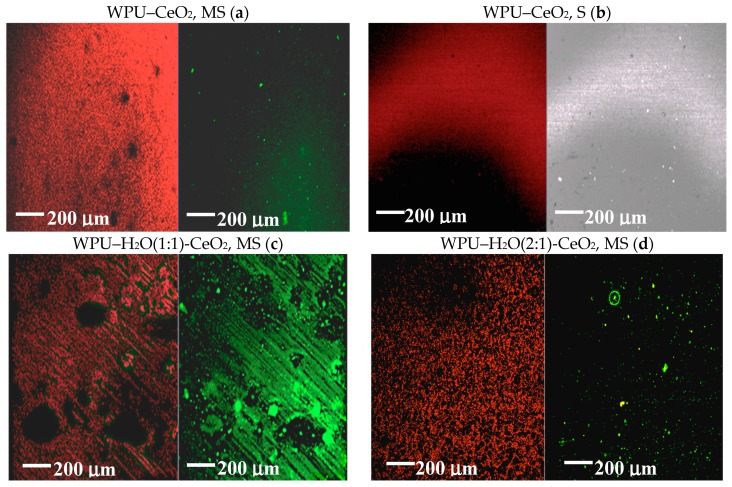
CLSM analysis observations on glass substrates of PU–CeO_2_ (5 wt %) hybrid coatings using different dispersion methods: (**a**) sonication (S); and (**b**) magnetic stirring (MS) during the polymerization process. The influence of water excess with respect to PU during the polymerization process using MS is also shown is also shown: (**c**) 1:1; and (**d**) 2:1.

As functional rare earth materials, cerium compounds have found wide applications in some important areas such as catalysis, electronic and chemical materials due to the specific 4f energy levels of the Ce element [35]. It is of fundamental importance to identify low cost, versatile, quick, straightforward and reproducible fabrication methods for coating application purposes [36,37,38]. Since the properties of nanostructured materials are strongly dependent on their shape and size, the dispersion of CeO_2_ nanostructures can play a key role in the performance of WPU–CeO_2_ hybrid coatings on aluminum alloys. In this regard, a first set of experiments was conducted in a 3 wt % NaCl solution to obtain information on the electrochemical performance of WPU–CeO_2_ hybrid coatings synthesized with 5 wt % of CeO_2_ nanostructures and dispersed for 1 h by magnetic stirring or sonication methods (Figure 1). Considering that this work evaluates the barrier properties of WPU–CeO_2_ hybrid organic–inorganic coatings, samples with different water-based excesses in the polyurethane content (1:1 and 1:2) were also analyzed in this figure. All the samples reached a steady state after 1200 s, although, in general, the *E*_ocp_ evolutions show very different shapes depending on the dispersion method and water quantity used during the polymerization process. The *E*_ocp_ shifted to a positive direction up to 850 mV_SCE_ for samples dispersed with sonication, in comparison with the uncoated specimens (−750 mV_SCE_) and specimens dispersed with magnetic stirring (*E*_ocp_ 200 mV_SCE_). On the contrary, samples with an excess of water during the polymerization process displayed similar potentials as that presented by bare aluminum. Pure WPU coatings displayed a stable potential-time dependence at ~−400 mV_SCE_ for a long period of time (300–3300 s), however, after 3300 s, the *E*_ocp_ presented clear fluctuations. It is well known that *E*_ocp_ does not provide detailed information on the corrosion kinetics but shows the electrode potential in the corrosive environment. In this way, *E*_ocp_ could indicate the possible occurrence of corrosion processes, i.e., a lower *E*_ocp_ indicates higher degradation probability whereas displacements in the positive directions suggest a slight inhibition. A visual inspection of coatings obtained with the magnetic stirring method showed that the presence of water in the reaction medium caused a porous morphology and agglomeration of ceria nanostructures, which possible resulted from the alcohol, amine and CO_2_ formation during the reactions between PU and water (Figure 2) [39]. The visual observation of porous morphology can compromise the coating performance, with less resistance to water penetration and possible aggressive species migration, which might explain the *E*_ocp_ evolutions. In order to corroborate this assumption, dispersion analyses on hybrid coatings was performed by CLSM and the results are shown in Figure 3a–d. CLSM is a common tool for the quantitative determination of film-core interfaces or dispersion evaluation, inclusion grain boundaries, among others [40,41,42,43,44,45,46,47,48]. The figures demonstrate that more uniform hybrid coatings can be obtained when CeO_2_ nanostructures are sonicated before being added to the polymer matrix. In agreement with theoretical works, it can be attributed to sound waves propagating into the aqueous medium, which causes mechanical tension over the electrostatic forces of attraction, i.e., van der Waals during sonication method [49,50].

It established that the dispersion of nanoparticles in the polymer matrix depends on many factors such as the interfacial nature of the nanostructures, the polymer nature and their intrinsic properties and how they are incorporated into the polymer matrix [51,52]. For hybrid coating application, high dispersion of nanoparticles is desired which it can seal the gas bubbles formed during a normal polymerization process; where water reacts with isocyanate groups producing CO_2_, which are encapsulated in the polymeric matrix. Additionally, it offers enhanced barrier properties against aggressive ion penetration. In this way, CeO_2_ particles can help to seal the gas bubbles that are formed during the WPU polymerization process, probably because the nanostructures tend to occupy the gas bubble sites [53].

**Figure 4:**
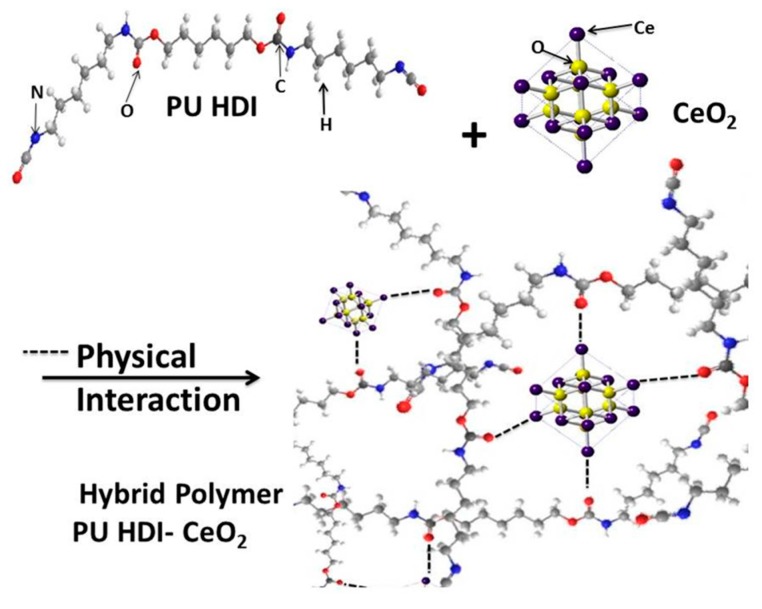
Physical interaction of nanoceria and WPU to form hybrid polymer.

On the other hand, as-prepared WPU coatings show that, when an excess of water is used, significant quantity of bubbles are formed during the polymerization reaction. Consequently, based on these experiments, it was concluded that a lower susceptibility to agglomeration of CeO_2_ nanostructures can be obtained with a stoichiometric water ratio in combination with the sonication approach. Therefore, all the results and discussion are confined to hybrid coatings obtained with the sonication method, as shown in Figure 4.

### 3.1. Structural and Optical Characterizations

**Figure 5:**
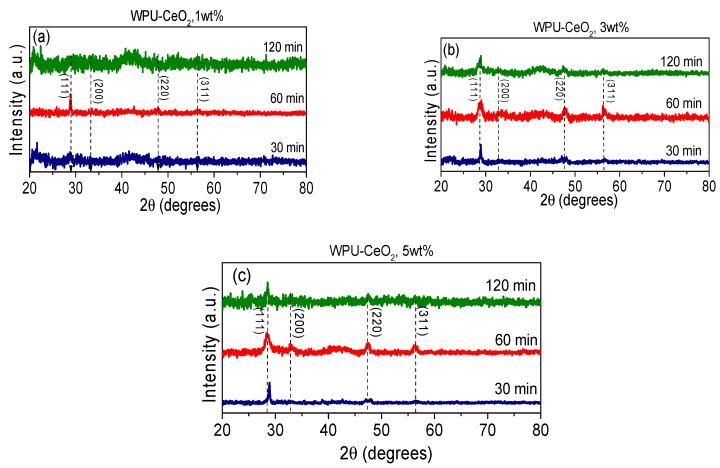
XRD patterns of WPU–CeO_2_ hybrid composites: (**a**) 1 wt % CeO_2_; (**b**) 3 wt % CeO_2_; and (**c**) 5 wt % CeO_2_.

The XRD patterns of the as-prepared organic–inorganic bulk composite are shown in Figure 5a–c. The XRD diffraction patterns display the crystallographic peaks corresponding to CeO_2_ in the θ–2θ range at 22.59°, 33.07°, 47.47° and 56.33° (PDF: 01-081-0792). It can be seen that all the diffraction peaks are broad and peak intensities are relatively weak, which is possibly due to the low content of CeO_2_ nanoparticles. The crystallite size was estimated using Scherrer’s equation for samples with higher CeO_2_ content (3 and 5 wt %) and the results are shown in Table 2 [54]. From these results, it can be seen that a balance in the energy supplied by the sonication approach is required to obtain a small crystal size, which is also dependent on the cerium content. For example, hybrid coatings synthesized with 5 wt % of CeO_2_ nanostructures and sonicated for 60 min have a crystallite size of 8.2 nm, whereas larger crystallite sizes (>20 nm) were obtained with 30 and 120 min of dispersion time. On the other hand, by adding 3 wt % of inorganic nanoparticles, a small crystallite size (8.9 nm) was also obtained with 60 min of sonication. It is generally known that the sonication time is critical to agglomeration and stability of nanoparticles. When sonication is carried out without interruption for longer periods of time, the nanoparticles tend to agglomerate more. This re-aggregation of the particles does not seem to be due to the sonication process by itself, but to the solution heating [55]. However, in the case of hybrid coatings, the stability and size of the CeO_2_ nanostructure must remain after being added to the polymer matrix and, in these cases [56,57,58], the crystallite size can reach a critical value some moments before polymerization begins. A key observation is that due to the solution heating, there is an optimum sonication time to refine the crystallite size and control the polymerization process. In agreement with a previous work, the dispersion of nanoparticles in the polymer matrix proceeds according to the following steps: (1) integration of CeO_2_ nanoparticles in water to the HDI; (2) dispersion of the largest particles into smaller ones, with more uniform nanocrystals; and (3) re-aggregation of the nanocrystals as the solution temperature rises.

**Figure 6:**
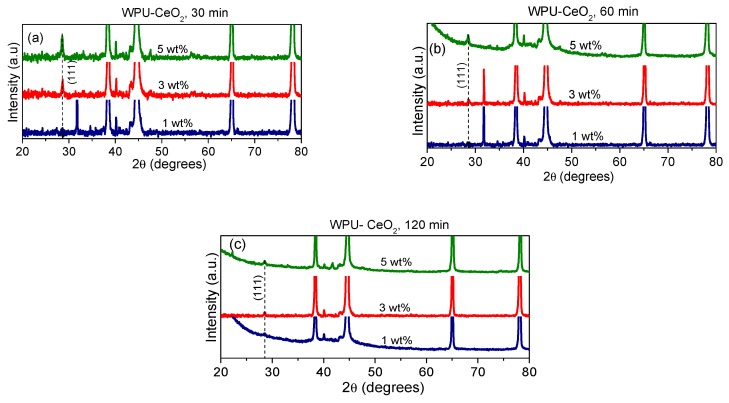
XRD patterns of different hybrid coatings deposited on AA7075: (**a**) 30 min; (**b**) 60 min; and (**c**) 120 min of sonication.

**Figure 7:**
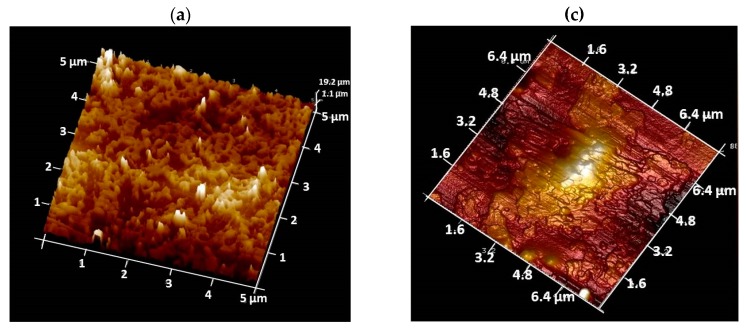

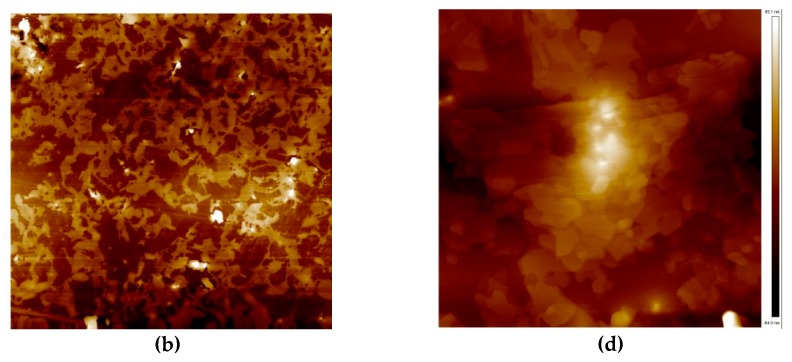
AFM image of two sonicated coatings: (**a**,**b**) WPU–CeO_2_, 30 min (3 wt %); and (**c**,**d**) WPU–CeO_2_ 60 min (5 wt %).

The structure of organic–inorganic hybrid coatings on the metallic substrates was also examined and the patterns are shown in Figure 6a–c. The high intensity peaks correspond to the reflections from the substrate using a Bragg–Brentano configuration and the intensity slightly decreases as the cerium oxide content increases. Preferential growth was observed during the coating deposition onto the metallic substrate, which depended on the sonication time and ceria quantity, although CeO_2_ peaks still match the face-centered cubic (fcc) structure Fm3m with a lattice parameter of 5.411 Å. This stability of the nanostructure after being added to the polymer matrix has been previously observed [50,55,57]. Nonetheless, variation in the crystallite size probably causes differences in the physical integration process, which in turn would affect barrier properties. AFM as well as other techniques such as grazing incidence X-ray diffraction (GIXRD) have been successfully used to confirm distribution of the nanostructures into the coatings along the thickness direction [59]. In this way, further support of the physical integration during the synthesis of hybrid coatings with 3 wt % (30 min) and 5 wt % (60 min) was performed by AFM examination of coatings, where topography aspects show that a 60-min dispersion prevented the formation of large agglomerates (Figure 7).

**Figure 8:**
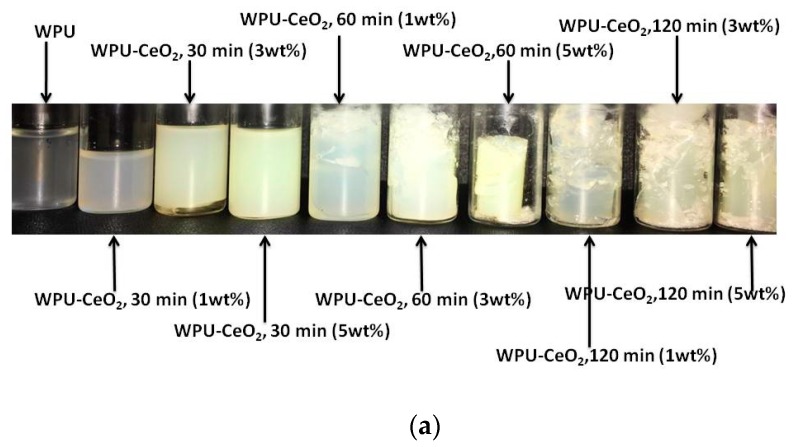

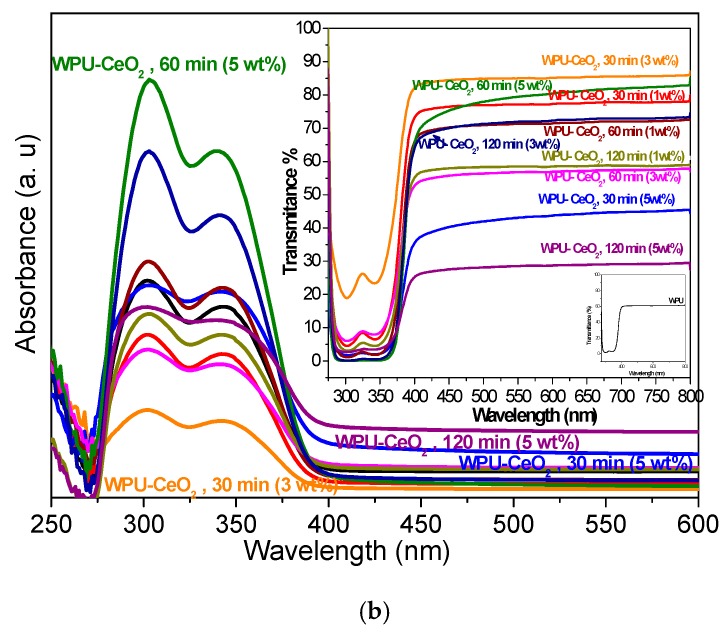
(**a**) Photographs of Visual aspects of the as-prepared samples after two years of polymerization and (**b**) UV-Vis spectra of water-based PU–CeO_2_ hybrid coatings using different amounts of nanostructures (1, 3 and 5 wt %) and sonication time (30, 60 and 120 min).

Figure 8 shows the visual aspects of the samples after two years of polymerization and UV-Vis spectra of WPU–CeO_2_ coatings on glass substrates. Two absorption peaks in the ultraviolet region can be observed at ~302 and ~342 nm, although no clear trend with the sonication time or amount of CeO_2_ nanostructures is obtained. The absorption peaks could be related to the charge transfer (2p O → 4f Ce) absorption edge of CeO_2_, which filled the well-known f to f spin-orbit splitting of the Ce 4f state [60,61], although some contributions from water base polyurethane were due to wavelengths shorter than 380 nm, where the π–π* (~300 nm) and n–π* (~330 nm) cannot discarded. The absorption values were also characterized by a strong reduction starting at a wavelength of 370 nm that extends up to the visible region. Interestingly, as-obtained samples with 5 wt % CeO_2_ and dispersed for 60 min displayed the highest values of absorbance in the UV region, but is also one of the most transparent coatings (82%). In fact, the WPU–CeO_2_, 60 min (1 wt %), PU–CeO_2_, 30 min (1 wt %) and PU–CeO_2_, 30 min (3 wt %) coatings all show transmittances above the PU coatings (>60%). From these, samples with 30 min (3 wt %) transmit about 86% (highest). These results demonstrate that under proper synthesis conditions the addition of nanoceria enhances the optical transparence of WPUs, which is one of the most important characteristics for coating applications (Figure 8a). As expected, the UV region confirms that CeO_2_ nanoparticles act as strong UV absorbers.

### 3.2. Electrochemical Performance

Commercial PU is commonly used for wood and metal finishing applications, giving brightness, toughness and wear resistance to materials. It is important to remark that CeO_2_ used as a coating or chemical conversion treatment has been frequently reported by different researchers using conventional techniques such as dip-coating, sol-gel, and more recently sputtering [62,63,64,65,66]. However, this work proposes a novel synthesis of hybrid coatings with ceria through reinforcing crosslinks during the curing process of water-based polyurethane. The barrier effect of the WPU–CeO_2_ hybrid coatings deposited on AA7075 was thereafter evaluated through several electrochemical parameters such as charge transfer resistance and capacitance. Non-destructive electrochemical techniques such as *E*_ocp_ and EIS were chosen because the performance of the hybrid system (WPU–CeO_2_/AA7075) can be continuously monitored for long term immersion.

Immersion tests were carried out in a 3% NaCl solution for 24 cycles. In each cycle, the following electrochemical techniques were performed in a sequence: *E*_ocp_ (900 s) and EIS. Selected results from cycles 1, 12 and 24 are discussed here to represent the early, middle and final stages of various hybrid coating systems in accelerated immersion testing.

**Figure 9:**
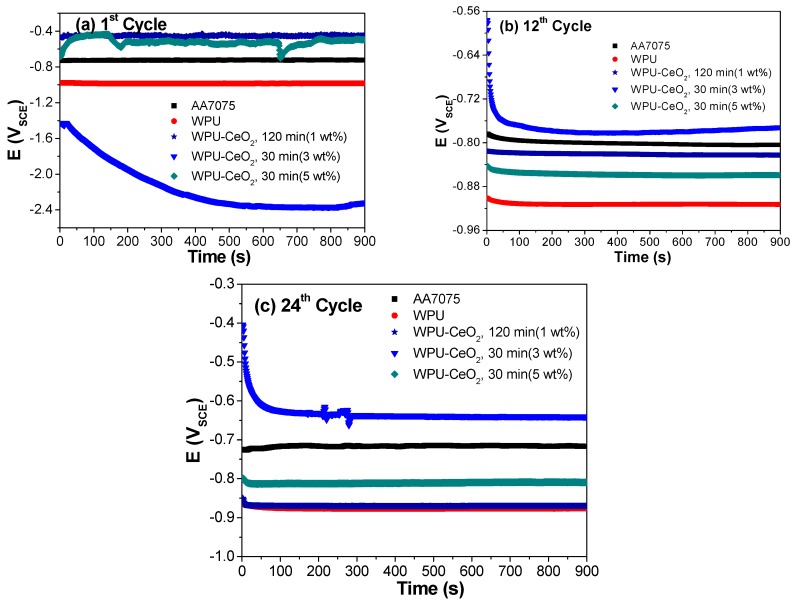
*E*_ocp_ measurements of cycles: (**a**) 1; (**b**) 12; and (**c**) 24 cycles of coated systems using a 3 wt % NaCl solution after 30, 60 and 120 min of sonication. For comparison purposes, the figures also present the *E*_ocp_ evolutions of pure PU and uncoated metallic substrates.

*E*_ocp_ evolutions of selected hybrid coatings in a 3% NaCl solution are shown in Figure 9a–c for cycles 1, 12 and 24, respectively. It can be seen from this figure that the OCP values reached the steady state quickly, normally within 100 s for most systems. Therefore, the *E*_ocp_ values at the end of 900 s were chosen as the steady state values and are summarized in Table 3 for 1, 6, 12, 18 and 24 electrochemical cycles. The *E*_ocp_ evolutions of bare metallic substrates and pure WPU coating on AA7075 are also presented for comparison. Bare AA7075 remained stable during the evaluated cycles with potential values varying from −0.71 to −0.80 V_SCE_. The classical behavior of shifting *E*_ocp_ values in the negative direction in the WPU/AA7075 system was also observed in the diagrams (−0.92 V_SCE_). On the other hand, the effect of sonication time for the incorporation of CeO_2_ nanostructures into the polymer matrix was clearly observed in the *E*_ocp_ measurements, where corrosion potentials increased with the sonication times. However, no clear trend can be found between *E*_ocp_ and the amount of ceria nanostructures. In general, the presence of the nanostructures in the polymer matrix caused an initial shift (1st cycle, C1) of *E*_ocp_ in the positive direction for most coatings. However, *E*_ocp_ eventually shifted to negative values for most coatings after long immersion (24th cycle, C24). The hybrid system of WPU–CeO_2_, 120 min (1 wt %) initially exhibited the most positive shift in *E*_ocp_, which was approximately 270 mV higher than that of bare AA7075. Corrosion potentials were significantly affected by the immersion time and after 12 cycles (C12), all the hybrid samples shifted to the opposite (more active) direction. From overall samples, hybrid coatings synthesized with 3 wt % ceria nanoparticles and 30 min of sonication displayed the largest positive displacement (~1.65 V) and reached a potential of −0.64 V_SCE_ at the end of 24 cycles.

The observed differences in the corrosion potentials can be explained as follows: a low concentration of ceria nanostructures (1 wt %) can be well dispersed in the polymer. Nonetheless, the quantity is low and therefore it is insufficient to interact appropriately in the crosslinking of PU during the curing process. On the contrary, 5 wt % of ceria seems to be a high amount, but the energy generated during the sonication process was not enough to keep the nanostructures well dispersed and agglomeration occurred. Therefore, the hybrid coating with 3 wt % of ceria and short sonication time (30 min) seems to be the optimal condition that yielded well-dispersed systems with sufficient nanostructures for crosslinking. It is also important to mention that it was not considered to perform further analyses with the sonication time (especially with 3 wt %) because the differences in the final potentials (not shown here) were very small, in the range of −0.6 V_SCE_ and −0.75 V_SCE_.

It is well known that an electrochemical process is related thermodynamically and kinetically to several important variables: *E*_ocp_, corrosion current density and/or resistance. Normally, the displacement of *E*_ocp_ to positive values is related to the increase in barrier properties while decreased barrier properties are obtained with the displacement towards negative values. Therefore, it is expected that coating samples with higher ennoblement in *E*_ocp_ should present an enhancement in the charge transfer resistance (lower corrosion current density). To corroborate this statement, EIS measurements were evaluated for 24 cycles (C24).

The EIS technique is one of the most used non-destructive techniques for researching the evolution of the behavior of materials in an aggressive environment, i.e., an analysis of the degradation with time. One of the most relevant applications of EIS is the evaluation of the properties of coatings deposited over metallic substrates. EIS allows the determination of different resistive contributions that participate in an electrochemical reaction. For example, when coatings are analyzed at high frequencies, it is possible to obtain important coating parameters such as coating resistance, coating defects (e.g., pores, cracks, and blistering) resistance. All of them in combination with coating capacitance characterize the barrier system. On the other hand, in the region of low frequencies, it is possible to obtain important information regarding metal reactions and charge transfer through the coating toward the metallic substrate, as well as the electrolyte resistance [1]. In this way, this technique gives kinetic information about the mechanism and half-life through interactions in the electrode–electrolyte interface.

**Figure 10:**
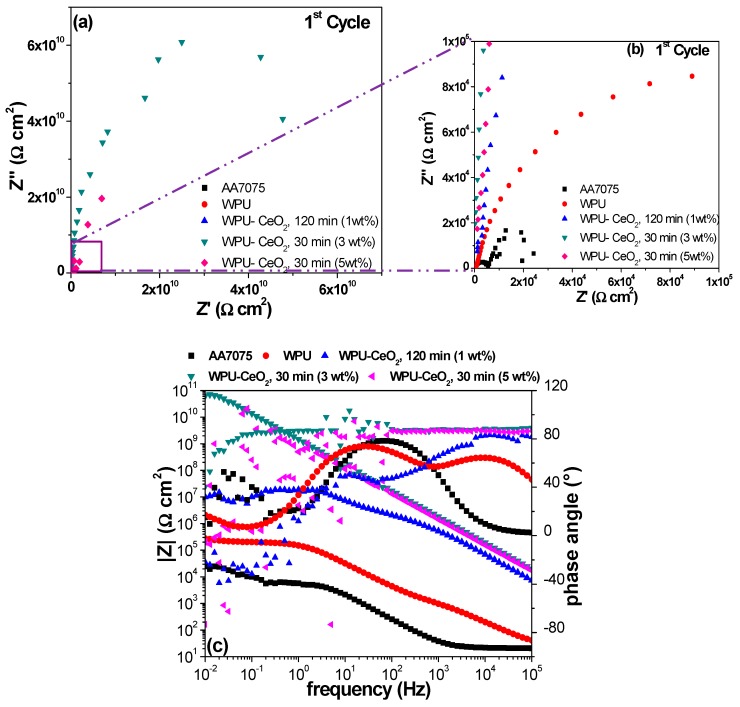
Impedance spectra for the electrodes coated with PU-CeO_2_ hybrid films of bare substrates in a 3 wt % NaCl solution after the first cycle of continuous immersion. (**a**) Nyquist plots; (**b**) Nyquist graphs magnification and (**c**) Bode diagrams.

**Figure 11:**
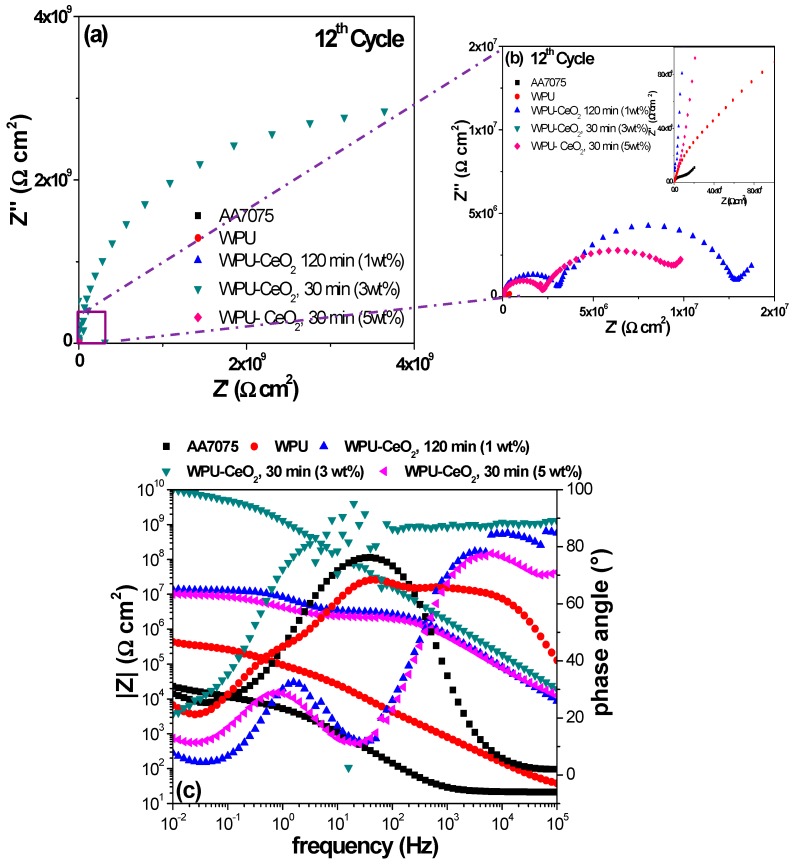
Impedance spectra for the electrodes coated with PU-CeO_2_ hybrid films of bare substrates in a 3 wt % NaCl solution after the twelve cycle of continuous immersion. (**a**) Nyquist plots; (**b**) Nyquist graphs magnification and (**c**) Bode diagrams.

**Figure 12:**
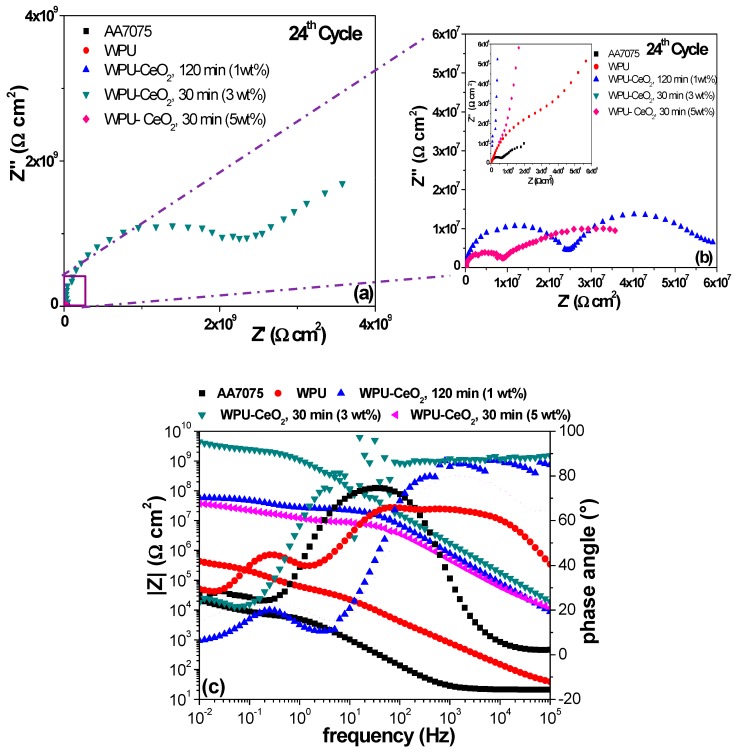
Electrochemical performance of the as-obtained samples after 24th cycle of evaluation in a 3 wt % NaCl solution. (**a**) Nyquist plots; (**b**) Nyquist graphs magnification and (**c**) Bode diagrams.

EIS measurements of the different systems were carried out for 24 cycles in continuous immersion. Figure 10, Figure 11 and Figure 12 show the EIS results in the representation of Nyquist and Bode plots for selected WPU–CeO_2_ hybrid coatings for three cycles: cycles 1, 12 and 24 represented early, middle and final stages, respectively. The electrochemical response of bare AA7075 and pure PU coated AA7075 are also presented for comparison. The EIS result of bare AA7075 after the first cycle shows two resistive contributions (Figure 10): one characteristic of the passive layer of aluminum oxide and the second related to the charge transfer resistance of the metallic substrate with an order of 3 × 10^4^ Ω cm^2^. Bode plots show that the phase angle started to form a second time constant in the frequency of 10^−2^ Hz. In the case of pure WPU coatings, two time constants were also observed. The first resistive contribution can be attributed to the organic coating pores. In this step, the aggressive medium penetrated the coating and reached a region of intact coating. The second semicircle is attributable to the resistance in the pore–electrolyte–intact coating interface. The poor impedance value of pure PU coating (~10^5^ Ω cm^2^) is probably a combination of the characteristics of WPU and the thin wet film thickness (100 μm) used in this work, in comparison with other researchers (200 μm) [63,67]. Nevertheless, the main goal of this research work is to determine the effect of added CeO_2_ nanostructures to a commercial WPU matrix and evaluate the electrochemical performance in a relatively short period of time, where the thickness of 100 μm was deemed as an adequate option.

As expected, the hybrid coatings showed an increase in the charge transfer resistance with respect to the pure WPU coating. The effect of ceria content and sonication time on the total impedance values can be seen based on selected hybrid coatings. Specifically, the hybrid coating using 1 wt % of nanostructures and 120-min sonication shows a total resistance in the order of ~10^7^ Ω cm^2^, which is about two orders of magnitude higher than that of the pure WPU coating. The electrochemical behavior of these samples also presented two resistive contributions corresponding to the coating porosity and the pore–electrolyte–intact coating interface. Dispersing 3 wt % of CeO_2_ in the polymer matrix for 30 min displayed significant improvement in the impedance modulus with one apparent semicircle, the coating resistance value is in the order of 10^11^ Ω cm^2^. Finally, the hybrid coating processed with 5 wt % of CeO_2_ (30 min) showed an intermediate value of total impedance, which is about 10^9^ Ω cm^2^. This coating showed a wide dispersion at low frequency regions, which may be due to the equipment limitation (~1 pA) or small current variations during the acquisition. The significant improvement of total resistance in the hybrid coating systems may be explained by the increase in film density as well as a higher network condensation with the presence of nanoceria [68,69].

The EIS results after 12 cycles (C12) are presented in Figure 11, where it can be observed that total impedance for bare AA7075 remains almost unchanged. A slightly increase in the impedance values of pure PU (8 × 10^5^ Ω cm^2^) may be due to the saturation stage, which occurs after water diffuses through micro-pores in the polymeric matrix or the formation of some corrosion products [70]. The hybrid coating with 1 wt % of CeO_2_ (120 min) also shows stabilization with similar values from the first cycle, although the two time constants are well-defined here. The hybrid coating with 3 wt % of ceria nanoparticles and 30 min of sonication shows a slight decrease in the total resistance (~1 × 10^10^ Ω cm^2^) in comparison with the 1st cycle result. However, this impedance magnitude still suggests excellent protection of the AA7075 substrate by this coating. In contrast, hybrid coatings synthesized with 5 wt % of ceria and a 30-min sonication showed a considerable reduction in the coating resistance, 1 × 10^7^ Ω cm^2^, which is close to 2–3 orders of magnitude decreased compared with the first cycle result. This significant reduction can be attributed to the poor dispersion of ceria nanostructures into the polyurethane matrix, provoking the formation of agglomerate areas and micro-pore areas, which provides pathways for electrolyte diffusing through the coating to the metal substrate (AA7075).

Figure 12 presents the EIS results from the last (24th) cycle, where it is seen that bare AA7075 began to experience a slight reduction in the total impedance 1 × 10^4^ Ω cm^2^. The total resistance for the pure WPU/AA7075 system continued to decrease (3 × 10^5^ Ω cm^2^). At this stage, the impedance value suggests that the saturation step of the WPU coating was possibly exceeded and diffusion of corrosive species to the metallic surface was likely. An important change occurred at the 24th cycle for the 1 wt % (120 min) and 5 wt % (30 min) hybrid coatings, which both showed an increase in the total resistance, ~7 × 10^7^ Ω cm^2^ and ~4 × 10^7^ Ω cm^2^, respectively. It is well known that cerium oxide tends to form a layer of stable oxide on the aluminum surface [71]. This oxide layer potentially seals the micro-pores or blocks pathways formed through the polyurethane coating, which will cause an increase in the total resistance through a self-regenerating mechanism of effective barriers. This process may explain the increasing total resistance in the latest stage of the immersion test. Finally, it is clear that an optimal electrochemical performance was achieved with 3 wt % of CeO_2_ nanoparticles sonicated for 30 min. This hybrid coating sample exhibits an impedance value about 4 × 10^9^ Ω cm^2^ after 24 cycles of immersion, which is significantly higher than all the other evaluated hybrid coatings. It is also important to mention that the Nyquist plots show the initial formation of two time constants, which is indicative of slight degradation of hybrid coatings, but without reaching the metallic surface.

The second time constant after 24 cycles confirmed the initiation of hybrid coating degradation after long immersion times, but the high total resistance value suggests excellent protection by this hybrid coating and possibly for a much longer time.

Impedance data of the hybrid coatings were analyzed by fitting them to an equivalent electrical circuit model. An equivalent circuit considers a combination of passive elements such as resistors, capacitors, inductors and different forms of impedance distributions, which should have a physical/electrochemical basis in the modeled system. Thus, an equivalent circuit model can be constructed to reflect the charge transfer path in the evaluated system and represents the electrochemical performance with precision. Therefore, a proposed equivalent circuit can be used to yield information on the corrosion mechanism and/or corrosion kinetics (corrosion rate). 

**Figure 13:**
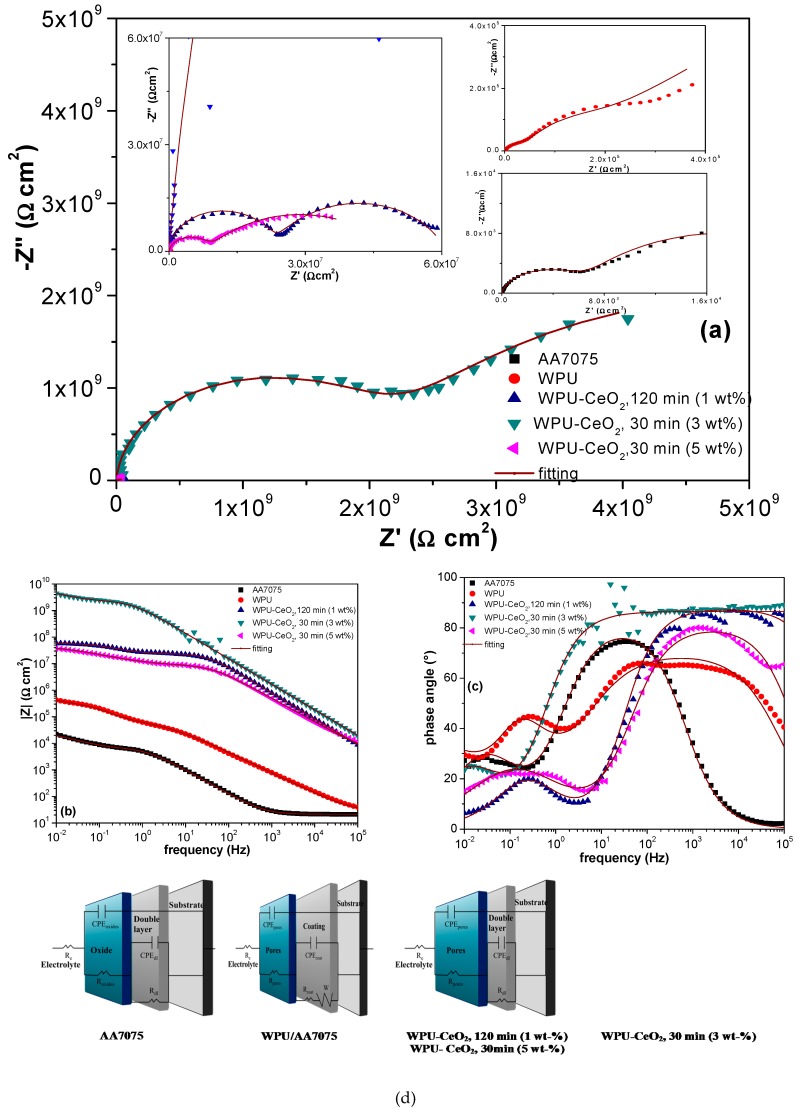
Impedance spectra of representative samples evaluated in a 3 wt % NaCl solution (**a**) Nyquist; (**b**) total impedance; (**c**) phase angle. This figure also shows (**d**) the equivalent circuits used to fit the experimental data (continuous line).

Figure 13 shows four equivalent circuit models for different systems and examples of Nyquist and Bode plots with their corresponding fitting. During simulation, a constant phase element (CPE) was used instead of an “ideal” capacitor to account for the deviations from ideal behavior. The impedance of a CPE (Z_CPE_) can be defined by Z_CPE_ = (1/*Y*)/(jω)*^a^*, where ω is frequency, *Y* is pseudo-capacitance and *a* is associated with the system homogeneity; when this equation describes a capacitor, *a* = 1 and *Y* = C (capacitance).

Various RC Elements are used to describe suitable components of the AA7075 alloy, pure PU and selected hybrid PU/CeO_2_ coatings (1 wt % 120 min, 3 wt % 30 min and 5 wt % 30 min) dispersed by sonication. 

In the equivalent circuits, *R*_e_ is the electrolyte resistance; CPE_oxides_ and *R*_oxides_ represent pseudo-capacitance and resistance of a naturally occurring crystalline form of Al_2_O_3_. The reaction between active aluminum and atmospheric oxygen results in a thin passive layer (~2–4 nm) on the metal surface. This layer protects the underlying metal from further oxidation. *R*_ct_ is the charge transfer resistance of the corrosion process. It occurs when the Al_2_O_3_ layer has been damaged and the electrolyte begins to reach the metal surface. CPE_dl_ is the pseudo-capacitance of the electrochemical double layer at the metal-electrolyte interface. In the equivalent circuit proposed for the AA7075 alloy coated with pure PU, CPE_pores_ and *R*_pores_ characterizes the pseudo-capacitance and resistance of pre-existing coating defects such as pores in organic coatings, which allow the initial permeation of electrolytes without reaching the metal substrate. CPE_coat_ and *R*_coat_ symbolize the pseudo-capacitance and resistance of the pure PU coating, which are in series with a Warburg element (*W*) accounting for diffusion of reactive species through the pores to the metal-coating interface. In the Nyquist plot, the diffusion Warburg element appears as a semicircle with a straight line with slope equal to 1. The coating resistance decreases by the diffusion of water and other dissolved species through the coating, which will result in an increase in the coating conductivity. The electrical circuits proposed for WPU–CeO_2_ hybrid coatings with 1 wt % (120 min) and 5 wt % (30 min) are quite similar to other equivalent circuits (Figure 13) with two time constants. Before significant coating degradation occurs, pseudo-capacitance CPE_pores_ and resistance *R*_pores_ are used to model the system, and when the pores are saturated with electrolyte, pseudo-capacitance of double layer CPE_dl_ and its resistance *R*_dl_ are utilized. The significant decrease in coating performance occurred in the 12th cycle (C12), probably due to the bad distribution and agglomeration of ceria nanoparticles in the polymer matrix under the experimental conditions. Despite the significant decrease in performance, these coatings still offer high protection against corrosion compared with a pure polyurethane coating. From these measurements, it was observed that, after 24 cycles (C24), the most prominent corrosion protection was obtained for the hybrid coating with 3 wt % of CeO_2_ (30 min), suggesting that this hybrid coating still maintained excellent barrier properties under the test conditions.

Selected fitting results are shown in Table 4. The fitted data were acquired considering a χ^2^ < 10^−3^. All the hybrid coatings displayed higher impedance values than pure WPU coatings and the higher polarization resistance obtained for samples with 3 wt % of CeO_2_ (8.35 × 10^9^ Ω cm^2^) was about four orders of magnitude higher than that of pure polyurethane coating (2.94 × 10^5^ Ω cm^2^). Under these conditions, the polarization resistance values are quite similar to other hybrid coatings using oil-based polyurethanes. The presence of an adequate amount of ceria nanoparticles combined with proper sonication times ensures that nanoparticles seal uniformly the porous polymer acting as a barrier to avoid electrolytic pathways across the coating volume; i.e., retarding the fast ingress of aggressive ions and consequently enhancing the electrochemical properties.

### 3.3. Salt Spray Chamber

The salt spray test was used to obtain complementary information on the hybrid coating performance by following the ASTM B117 standard [34]. The salt fog exposure commonly provides a controlled corrosive environment to acquire corrosion resistance information of coated metals in a simulated natural environment. This method has been widely utilized to evaluate non-chromate chemical conversion coatings (Ti/Zr/Mn/Mo) [72], cerium-based inhibitors [73] and different hybrid coatings (sol-gel hybrid coatings based on Si and Zr) [74] and nano ZnO deposited on WPU coatings [75] on an AA7075 alloy. The exposure time was varied from 48 to 1080 h depending on the visual damage.

**Figure 14:**
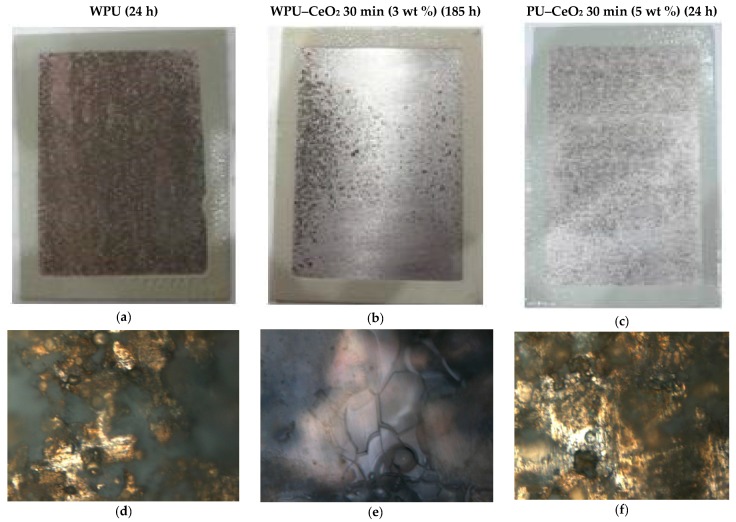
Salt fog tests (5 wt % NaCl) with their corresponding surface analysis by light microscopy of selected samples: (**a**,**d**) WPU/AA7075, 24 h; (**b**,**e**) WPU–CeO_2_/AA7075 (3 wt %, 30 min, 185 h); and (**c**,**f**) WPU–CeO_2_/AA7075 (5 wt %, 30 min, 24 h).

In order to properly compare different systems, the dry film coated on the metallic substrate was close in thickness: PU (32.74 μm); WPU–CeO_2_, 30 min (3 wt %, 33.92 μm); and WPU–CeO_2_, 30 min (5 wt %, 30.41 μm). The influence of two parameters, weight fraction of nanoparticles in the coatings and sonication time, were analyzed in the salt spray tests. Selected specimens with different ceria contents were exposed in a monitored salt spray chamber for up to 185 h. Microscopic images of selected samples after exposure are shown in Figure 14a–c. In agreement with 24-h electrochemical measurements, the salt spray results show that the corrosion resistance was quite influenced by the nanostructure content in the polymer matrix as well as sonication time. An optimal condition was reached with 3 wt % of ceria nanoparticles dispersed for 30 min in the WPU matrix (Figure 14b). Higher CeO_2_ contents (e.g., 5 wt %) caused an increase in corrosion rate (Figure 14c). In fact, samples with 1 wt % and 5 wt % of nanostructures began to show visual signs of corrosion within 24 h of exposure, which is similar to that of pure WPU coated samples (Figure 14a).

When all the samples were inspected after 24 h of exposure, the hybrid coating WPU–CeO_2_ (3 wt % 30 min) did not show rust, indicating that good corrosion protection had been achieved with proper ceria content in the polymer matrix in combination with proper sonication time. In contrast, the corrosion process started to appear on WPU/AA7075, WPU–CeO_2_/AA7075 (both 1 wt %-120 min and 5 wt %-30 min) within the first hours. More and more rust was observed up to the polymeric matrix as the exposure time increased (48 h), which is reasonable taking into account that the electrochemical process proceeds with time. According to the ASTM G46 standard [73], samples with 1 wt % and 5 wt % of ceria showed a rate of pits classified with a density of 3 (1 × 10^5^/m^2^) and a size of 1 (0.5 mm^2^), whereas hybrid coatings with optimal conditions displayed this classification after 185 h of exposure. 

The micrographs also show that even with low resolution and depth of optical light microscopy, surface damage can be clearly seen in the evaluated systems. In the case of the pure WPU coating, there was a significant quantity of pits with salt around them after 24 h. A minor quantity was observed with ceria content of 5 wt % (30 min) in the polymer matrix after this time of evaluation. However, it clearly shows that this coating degraded after this exposure time and corrosive species reached the substrate. On the other hand, the hybrid sample containing 3 wt % ceria (30 min) shows some areas that were still covered by the hybrid coatings and others with small pits after 185 h of exposure.

The salt fog results mentioned above provided important information about the anticorrosion performance of the WPU–CeO_2_ self-healing coatings. Nevertheless, it has been recognized that there are some shortcomings for this test [74]. This test provides only qualitative evaluations and systematic errors could occur. For example, the specimens were manually sealed before exposure to salt fog. Although a standard ASTM procedure was strictly followed, it is still impossible to ensure that all the seals were exactly the same on each specimen. The specimen position in the salt spray chamber and so on could also cause errors. Despite these limitations, the salt spray tests matched well with short term electrochemical assessments and confirmed that by optimizing the synthesis parameters, WPU–CeO_2_ hybrid coatings could provide excellent corrosion protection to AA7075 substrates.

Finally, the obtained results indicate that yellowing of WPU can be delayed with proper nanoceria content and at the same time offers an improvement in the corrosion resistance for the AA7075 aluminum alloy. This is possibly due to the electron flow being reduced by filling pores during the polymerization process, which in turn restrict the movement of corrosion species in the metal coating interface, preventing the common aluminum corrosion reactions;

(1) $$Al\to Al^{3+}+3e^{-},$$

(2) $$Al^{3+}+3H_{2}O\to Al{\left(OH\right)}_{3}+3H^{+}$$

(3) $$2Al{\left(OH\right)}_{3}\to Al_{2}O_{3}+3H_{2}O$$

(4) $$Al^{3+}+Cl^{-}\to Al{\left(Cl\right)}_{3}$$

## 4. Conclusions

Different amounts of CeO_2_ were incorporated into a commercial water-based polyurethane (PU-HDI) matrix to form hybrid coatings that were applied to both glass and AA7075 aluminum alloy substrates. Based on these results, the following conclusions can be drawn:

- Ceria nanoparticles dispersion in WPU follows the typical dispersion mechanism, where agglomeration can be avoided or minimized using the sonication approach.
- The excess of water in the reaction medium provokes important changes in the coating porosity that compromises its performance in terms of aggressive species diffusion through the WPU–CeO_2_/AA7075 systems.
- The fcc structure that remains after WPU polymerization indicates that CeO_2_ nanostructures are unaffected during their incorporation into the polymeric matrix, although the amount of nanostructures and sonication time significantly affect the average crystallite size (from 8.2 to 27.7 nm) and orientation onto the metallic substrates.
- The texture of CeO_2_ can be modulated with the synthesis parameters as well as affect the physical integration to the polymer matrix.
- The nanoceria amount and sonication time during the synthesis play a crucial role in enhancing the optical transparence of WPUs, which helps to delay yellowing.
- The electrochemical performance of the samples indicates that 1 wt % of CeO_2_ nanostructures is insufficient for a homogeneous incorporation to the polymers to seal pores while excessive nanostructures (e.g., 5 wt %) provoke agglomeration and coalescence in the polymer matrix.
- An optimal electrochemical performance is achieved with 3 wt % of CeO_2_ (30 min) nanoparticles, highlighting the importance of both ceria content and sonication time, as synthesis parameters.
- Hybrid coatings with optimal conditions displayed impedance values up to four orders of magnitude higher than that obtained for pure WPU on metallic substrate, which were in agreement with salt fog exposure tests.

## Figures and Tables

**Table 1:** Nominal composition of AA7075 aluminum alloy.

Element	Si	Fe	Cu	Mn	Zn	Ti	Cr	Mg	Al
Weight %	0.137	0.262	1.629	0.062	5.36	0.045	0.20	2.26	balance

**Table 2:** Crystallite size for WPU–CeO_2_ hybrid bulk composites.

Coating on AA7075	Crystallite size (nm)
WPU–CeO_2_, 30 min (3 wt %)	25.2
WPU–CeO_2_, 30 min (5 wt %)	27.7
WPU–CeO_2_, 60 min (3 wt %)	8.9
WPU–CeO_2_, 60 min (5 wt %)	8.2
WPU–CeO_2_, 120 min (3 wt %)	8.5
WPU–CeO_2_, 120 min (5 wt%)	22.4

**Table 3:** *E*_ocp_ measurements of hybrid organic–inorganic coatings on AA7075 aluminum alloys after 30, 60 and 120 min of sonication (C1: 1st cycle).

Sample	*E*_ocp_ (V vs. SCE)				
	C1	C6	C12	C18	C24
**AA7075**	−0.73 ± 0.01	−0.72 ± 0.01	−0.77 ± 0.03	−0.78 ± 0.03	−0.74 ± 0.05
**PU**	−0.80 ± 0.15	−0.80 ± 0.12	−0.76 ± 0.12	−0.84 ± 0.06	−0.82 ± 0.05
**PU–CeO_2,_ 30 min (1 wt %)**	−0.79 ± 0.1	−0.81 ± 0.09	−0.88 ± 0.09	−0.88 ± 0.08	−0.87 ± 0.11
**PU–CeO_2_ 30 min (3 wt %)**	−1.92 ± 0.78	−0.87 ± 0.2	−0.67 ± 0.01	−0.66 ± 0.04	−0.65 ± 0.07
**PU–CeO_2_ 30 min (5 wt %)**	−0.56 ± 0.12	−0.82 ± 0.1	−0.85 ± 0.06	−0.87 ± 0.06	−0.9 ± 0.08
**PU–CeO_2_ 60 min (1 wt %)**	−0.72 ± 0.02	−0.75 ± 0.04	−0.75 ± 0.03	−0.79 ± 0.07	−0.77 ± 0.02
**PU–CeO_2_ 60 min (3 wt %)**	−0.74 ± 0.07	−0.74 ± 0.04	−0.76 ± 0.02	−0.8 ± 0.07	−0.82 ± 0.08
**PU–CeO_2_ 60 min (5 wt %)**	−0.67 ± 0.06	−0.65 ± 0.11	−0.77 ± 0.06	−0.78 ± 0.05	−0.78 ± 0.04
**PU–CeO_2_ 120 min (1 wt %)**	−0.46 ± 0.01	−0.71 ± 0.02	−0.85 ± 0.02	−0.88 ± 0.02	−0.86 ± 0.02
**PU–CeO_2_ 120 min (3 wt %)**	−0.63 ± 0.02	−0.63 ± 0.01	−0.66 ± 0.01	−0.66 ± 0.02	−0.72 ± 0.02
**PU–CeO_2_ 120 min (5 wt %)**	−0.69 ± 0.04	−0.71 ± 0.06	−0.74 ± 0.02	−0.76 ± 0.02	−0.77 ± 0.01

**Table 4:** EIS fitting analysis of bare AA7075, WPU pure and WPU–CeO_2_ hybrid coatings by 1, 3 and 5% of CeO_2_ and 30 and 120 min of sonication.

Sample	*R*_s_ (Ω cm^2^)		*R*_coat_ (Ω cm^2^)	*R*_ct_ (Ω cm^2^)	*Y*_0coat_ (S*s^n^)	*n*_coat_	*Y*_0dl_ (S*s^n^)	*n*_tc_	*W* (S*s^1/2^)	χ^2^
AA7075		21.3	6.80 × 10^3^	2.24 × 10^4^	2.28×10^−5^	0.91	3.65 × 10^−4^	0.77	---	1.81 × 10^−3^
WPU		19.7	2.19 × 10^5^	7.52 × 10^4^	1.84 × 10^−6^	0.75	1.05 × 10^−9^	0.90	8.218	1.52 × 10^−5^
WPU–CeO_2_, 120 min (1 wt %)		45.2	2.35 × 10^7^	3.75 × 10^7^	2.73 × 10^−10^	0.97	3.17 × 10^−8^	0.76	---	1.8 × 10^−2^
WPU–CeO_2_, 30 min (3 wt %)		30.1	2.25 × 10^9^	6.10 × 10^9^	1.32 × 10^−10^	0.96	2.24 × 10^−9^	0.71	---	2.3 × 10^−2^
WPU–CeO_2_, 30 min (5 wt %)		50.6	9.32 × 10^6^	3.60 × 10^7^	9.47 × 10^−10^	0.87	6.93 × 10^−8^	0.62	---	1.1 × 10^−2^
